# Supplementary material for: A new perspective and assessment measure for common dissociative experiences: ‘Felt Sense of Anomaly’
Source: PLoS One. 2021 Feb 24;16(2):e0247037. doi: 10.1371/journal.pone.0247037 (PMC7904139; doi:10.1371/journal.pone.0247037)
Supplement: S1 File — (DOCX) [file pone.0247037.s001.docx]

**Supporting information 2:**

**Černis Felt Sense of Anomaly (ČEFSA) Scale**

Please read the following items and rate how often you have experienced these over the past **TWO WEEKS** using the following rating:

| 0 | 1 | 2 | 3 | 4 |
| --- | --- | --- | --- | --- |
| Never | Rarely | Sometimes | Often | Always |

**Please note that this should NOT be whilst under the influence of drugs, alcohol or legal highs.**

|  |  | Never | Rarely | Sometimes | Often | Always |
| --- | --- | --- | --- | --- | --- | --- |
| i | Things seem strange. | 0 | 1 | 2 | 3 | 4 |
| ii | I feel odd. | 0 | 1 | 2 | 3 | 4 |
| iii | Things seem weird. | 0 | 1 | 2 | 3 | 4 |
| iv | I feel surreal. | 0 | 1 | 2 | 3 | 4 |
| v | My experiences seem peculiar. | 0 | 1 | 2 | 3 | 4 |
|  | | | | | | |
| 1 | I feel like a stranger to myself. | 0 | 1 | 2 | 3 | 4 |
| 2 | I feel detached from my physical body (or parts of it). | 0 | 1 | 2 | 3 | 4 |
| 3 | Places that I know seem unfamiliar. | 0 | 1 | 2 | 3 | 4 |
| 4 | I don’t fully experience emotions. | 0 | 1 | 2 | 3 | 4 |
| 5 | I feel disconnected from the world around me. | 0 | 1 | 2 | 3 | 4 |
| 6 | I’m absorbed in my own world and don’t notice what is happening around me. | 0 | 1 | 2 | 3 | 4 |
| 7 | I feel like other people aren’t real. | 0 | 1 | 2 | 3 | 4 |
| 8 | My personality changes seemingly at random. | 0 | 1 | 2 | 3 | 4 |
| 9 | My body (or parts of it) feels like it doesn’t belong to me. | 0 | 1 | 2 | 3 | 4 |
| 10 | Familiar sights, smells (etc.) feel unfamiliar to me. | 0 | 1 | 2 | 3 | 4 |
| 11 | I can’t feel emotions | 0 | 1 | 2 | 3 | 4 |
| 12 | I feel disconnected from other people. | 0 | 1 | 2 | 3 | 4 |
| 13 | I find myself drifting off into my own world when I’m with others. | 0 | 1 | 2 | 3 | 4 |
| 14 | The world seems like it is fake. | 0 | 1 | 2 | 3 | 4 |
| 15 | I feel like I don’t have a personality | 0 | 1 | 2 | 3 | 4 |
|  |  | Never | Rarely | Sometimes | Often | Always |
| 16 | My body (or parts of it) feels unreal or strange. | 0 | 1 | 2 | 3 | 4 |
| 17 | People around me seem different or altered. | 0 | 1 | 2 | 3 | 4 |
| 18 | I feel detached from my emotions. | 0 | 1 | 2 | 3 | 4 |
| 19 | I feel as if I’m experiencing life from very far away. | 0 | 1 | 2 | 3 | 4 |
| 20 | I don’t notice how much time passes. | 0 | 1 | 2 | 3 | 4 |
| 21 | The world around me seems unreal. | 0 | 1 | 2 | 3 | 4 |
| 22 | I act like someone else without meaning to. | 0 | 1 | 2 | 3 | 4 |
| 23 | My body feels like it’s not under my control. | 0 | 1 | 2 | 3 | 4 |
| 24 | People I know seem unfamiliar. | 0 | 1 | 2 | 3 | 4 |
| 25 | I feel disconnected from my emotions. | 0 | 1 | 2 | 3 | 4 |
| 26 | The things happening around me seem unreal to me – like a dream or a movie. | 0 | 1 | 2 | 3 | 4 |
| 27 | I lose track of my surroundings. | 0 | 1 | 2 | 3 | 4 |
| 28 | I feel as though other people stop existing when I can’t see them. | 0 | 1 | 2 | 3 | 4 |
| 29 | I feel like I’m more than one person. | 0 | 1 | 2 | 3 | 4 |
| 30 | My body feels numb. | 0 | 1 | 2 | 3 | 4 |
| 31 | Things I’ve done many times before seem new or unfamiliar. | 0 | 1 | 2 | 3 | 4 |
| 32 | My emotions don’t seem real. | 0 | 1 | 2 | 3 | 4 |
| 33 | I feel detached from what I’m doing. | 0 | 1 | 2 | 3 | 4 |
| 34 | I feel like an alien or a ghost. | 0 | 1 | 2 | 3 | 4 |
| 35 | I freeze, unable to do anything. | 0 | 1 | 2 | 3 | 4 |

| **Factor** | **Item numbers** | **Factor** | **Item numbers** |
| --- | --- | --- | --- |
| Felt Sense of Anomaly | i + ii + iii + iv + v | Anomalous Experience of Emotion | 4 + 11 + 18 + 25 + 32 |
| Anomalous Experience of the Self | 1 + 8 + 15 + 22 + 29 | Altered Sense of Connection | 5 + 12 + 19 + 26 + 33 |
| Anomalous Experience of the Body | 2 + 9 + 16 + 23 + 30 | Altered Sense of Agency | 6 + 13 + 20 + 27 + 35 |
| Altered Sense of Familiarity | 3 + 10 + 17 + 24 + 31 | Altered Sense of Reality | 7 + 14 + 21 + 28 + 34 |

**Scoring:** total score. There are no reverse items.
Felt Sense of Anomaly scale (items i – v) may be used independently if a brief scale is required.
