# Supplementary material for: A new perspective and assessment measure for common dissociative experiences: ‘Felt Sense of Anomaly’
Source: PLoS One. 2021 Feb 24;16(2):e0247037. doi: 10.1371/journal.pone.0247037 (PMC7904139; doi:10.1371/journal.pone.0247037)
Supplement: Attachment — Submitted filename: Evaluations.docx [file pone.0247037.s002.docx]

Evaluations
1. In general, how do you rate the degree to which the paper is easy to follow and its logical flow? Good

2. Do the title and abstract cover the main aspects of the work? Yes

3. If relevant, are the methods are clear? No

Study design, ethical issues, and data analysis are mixed. Data were collected through social media (Facebook). Most of the time, Facebook users are uses by hiding or changing their correct name. So, what you say on quality of data, the real individuals these participated in your study? And so on… Generally, might be individuals are use fake account for Facebook. Clarify

4. If relevant, did the authors, make the underlying data available to the readers? Yes

5. Are the tables clear and legible? No

All tables’ titles are not self-explanatory. Rewrite all the tables as they are self-explanatory and easily understandable.

6. Does the paper raise any ethical concerns? Yes

7. Are the figure clear and legible? No

Title of the figure, rewrite under the figure and as scientifically sounded.

8. Most literatures review has no references. Please check it through the documents.

9. In table 6, you were listed gender (other), ethnicity (other). What is other?

10. Acknowledgements are too long.

11. I have not seen the importance of separating references of table 2 from the rests.

12. In table 6, what does it means by “First Episode Psychosis and Other psychosis disorder?” it is not clear for the readers especially by DSM-5, it has no meaning?

13. In table 6, you summarized that the age range =(18-74), mean = (41.51), and SD= (12.35). Did you check for normal distribution of the age? Think more about it. I think it is not appropriate. Same comment for Černis Felt Sense of Anomaly in the table 6.
