# Supplementary material for: A new perspective and assessment measure for common dissociative experiences: ‘Felt Sense of Anomaly’
Source: PLoS One. 2021 Feb 24;16(2):e0247037. doi: 10.1371/journal.pone.0247037 (PMC7904139; doi:10.1371/journal.pone.0247037)
Supplement: Attachment — Submitted filename: Cernis response.docx [file pone.0247037.s003.docx]

The language of the abstract is very convoluted. After rereading the “Definition and Framework Development section” several times, I think the abstract should be rewritten.

They talk about a “common denominator” and a construct” and don’t say from the outset that it was a Felt Sense of Anomaly (FSA) that they were looking to delineate from the beginning, based on their previous research. This convoluted language implies that FSA was discovered in the data that forms the basis of this paper.

Also, discussion of “common denominator” contradicts the concept of “discrete subset”. “Common denominator” suggests that they aim to find the one factor that underlies all of dissociative phenomena. The use of the term “common denominator” should be accompanied by the qualification that it is a common denominator of a subset of dissociative experience.

I suggest the following revision of ideas to the background and methods sections of the abstract (using phrases from the Definition and Framework Development section):

**Background**

The term ‘dissociation’ has long been argued to lack conceptual clarity and may describe several distinct phenomena. Based on our previous qualitative research into the lived experience of dissociation in people with psychosis diagnoses, we found that dissociation is commonly experienced as a subjective FSA. We therefore aimed to conceptualise and empirically delineate this discrete subset of dissociative experiences and develop a corresponding assessment measure.

**Methods**

First, a systematic review of existing measures was carried out to identify themes amongst dissociative experiences, including FSA phenomena. Second, item generation for the new measure was based on this literature review and our previous qualitative study. Third, measure development was achieved using exploratory (EFA) and confirmatory (CFA) factor analysis of 8861 responses to an online self-report survey. Fourth, the resulting measure was then validated via CFA with data from 1031 NHS patients with psychosis diagnoses.

It would also assist with the transparency of their goals if they discussed their qualitative study “Describing the indescribable” in the Introduction section. Most authors describe the evolution of their ideas that led to the current paper by reviewing their own publications in the Introduction.

Thank you for redoing Table 2 with a column that describes the sample more completely. Now that I realize that your goal in the review was more to find FSA phenomena in the literature rather than find one common denominator for all of dissociative phenomena, I think describing PTSD in the samples reviewed is unnecessary and can be deleted.

I appreciate the discussion of the relationship of Dissociative Amnesia to FSA.
