# Supplementary material for: A new perspective and assessment measure for common dissociative experiences: ‘Felt Sense of Anomaly’
Source: PLoS One. 2021 Feb 24;16(2):e0247037. doi: 10.1371/journal.pone.0247037 (PMC7904139; doi:10.1371/journal.pone.0247037)
Supplement: Attachment — Submitted filename: Response to Reviewers.docx [file pone.0247037.s004.docx]

Department of Psychiatry

Oxford University

Warneford Hospital

Oxford, OX3 7JX

Tel: 01865 618283

Email: emma.cernis@psych.ox.ac.uk

14^th^ January 2021

Dear Dr Sar,

**Re: *A new perspective and assessment measure for common dissociative experiences: ‘Felt Sense of Anomaly’.***

Our team again extend our thanks to the two reviewers for their kind comments and thoughtful feedback on the above manuscript.

Please find detailed below our responses to these, as requested in your email dated 11^th^ January 2021.

We very much hope that following the changes made to the manuscript - as outlined below - this manuscript now meets the standard required for publication in *PLOS One.*

Yours sincerely,

Dr Emma Černis

**Wellcome Trust Clinical Doctoral Fellow & Clinical Psychologist**

**Reviewer: 1**

1. Does the title give clear idea about the article? Yes

*No author response required*

2. Does the abstract concisely describe the content and scope of the project and identifies the project’s objective, its methodology and its findings, conclusions, or intended results? No
Under background, add something about dissociation more or its impact.

We thank the reviewer for this suggestion. We have added mention of how dissociation occurs across many mental health conditions under background as requested (within the constraints of the word limit).

3. Does the introduction give clear idea about the article? YES
Under introduction clarify “ […]”

Here, we believe the reviewer is referring to the opening quotation from Arntz (2013). The use of “[…]” is an ellipsis, which is the standard notation to denote the shortening of text.

4. Did method part is clear? No
Well, if specified who (author/s) were extracted data.

In the systematic review section we have now clarified that the first author carried out the data search and extraction.

What do you say about data quality assessment? Because, Participants were recruited via social media.

The reviewer rightly highlights the limitations of data quality arising from recruitment via social media. This is discussed in the latter part of the Discussion section.

Is all original paper report standard error? If no, what is your action? If yes, please specify.

We believe that this final comment is referring to the standard deviations in Table 2. Where these were not possible to retrieve from the original papers, these were not stated. However, the vast majority of papers that stated a mean also stated a standard deviation.

5. Are results clear and appropriate with title? Yes

*No author response required*

6. Revise the references as per the journal guideline

We are satisfied that the references for the main text of the manuscript are presented according to the journal’s guidelines.

7. The paper needs an English language copy editing from the beginning to the end. Please focus on it.

Our Oxford team have reviewed the language and are very confident of its grammatical correctness.

8. Generally, the paper is interesting. The methodology part needs revision.

We thank the reviewer for this comment. With regards to the methods section, please see point 4.

**Reviewer: 2**

The language of the abstract is very convoluted. After rereading the “Definition and Framework Development section” several times, I think the abstract should be rewritten.

We have considered this comment seriously as a team and have reworded the abstract.

They talk about a “common denominator” and a construct” and don’t say from the outset that it was a Felt Sense of Anomaly (FSA) that they were looking to delineate from the beginning, based on their previous research. This convoluted language implies that FSA was discovered in the data that forms the basis of this paper.

We thank the reviewer for this feedback. However, it was really not our intention in this study to confirm a construct from a previous study. The temporal sequence of the work was different to that (and the order of paper publication could not reflect this). The study described in this manuscript and the aforementioned qualitative study actually took place concurrently. At the time of the systematic review and development of the FSA matrix, very few of the qualitative interviews had taken place. Whilst it is possible that the emerging themes within the first interviews informed the interpretation of the systematic review, this is perhaps better described as a form of triangulation than confirmation. The concept of FSA was therefore independently established in this dataset without prior hypothesis – we really did have an open mind as a team during this work. As a result, we have not amended this language in the manuscript, and we hope that this explanation is satisfactory to the reviewer.

Also, discussion of “common denominator” contradicts the concept of “discrete subset”. “Common denominator” suggests that they aim to find the one factor that underlies all of dissociative phenomena. The use of the term “common denominator” should be accompanied by the qualification that it is a common denominator of a subset of dissociative experience.

We agree and thank the reviewer for highlighting this potential source of confusion. The text has been checked and amended where necessary to clarify this point.

I suggest the following revision of ideas to the background and methods sections of the abstract (using phrases from the Definition and Framework Development section):

**Background**

The term ‘dissociation’ has long been argued to lack conceptual clarity and may describe several distinct phenomena. Based on our previous qualitative research into the lived experience of dissociation in people with psychosis diagnoses, we found that dissociation is commonly experienced as a subjective FSA. We therefore aimed to conceptualise and empirically delineate this discrete subset of dissociative experiences and develop a corresponding assessment measure.

**Methods**

First, a systematic review of existing measures was carried out to identify themes amongst dissociative experiences, including FSA phenomena. Second, item generation for the new measure was based on this literature review and our previous qualitative study. Third, measure development was achieved using exploratory (EFA) and confirmatory (CFA) factor analysis of 8861 responses to an online self-report survey. Fourth, the resulting measure was then validated via CFA with data from 1031 NHS patients with psychosis diagnoses.

Please see above. We have reworded the abstract.

It would also assist with the transparency of their goals if they discussed their qualitative study “Describing the indescribable” in the Introduction section. Most authors describe the evolution of their ideas that led to the current paper by reviewing their own publications in the Introduction.

Please see our response to the reviewer’s second comment, above.

Thank you for redoing Table 2 with a column that describes the sample more completely. Now that I realize that your goal in the review was more to find FSA phenomena in the literature rather than find one common denominator for all of dissociative phenomena, I think describing PTSD in the samples reviewed is unnecessary and can be deleted.

We thank the reviewer for this feedback, and have amended Table 2 accordingly.

I appreciate the discussion of the relationship of Dissociative Amnesia to FSA.

We thank the reviewer again for this helpful suggestion and are pleased that our amendments have answered their previous concerns in this regard.
